# ChatGPT in surgery: a revolutionary innovation?

**DOI:** 10.1007/s00595-024-02800-6

**Published:** 2024-02-29

**Authors:** Mustafa Bektaş, Jaime Ken Pereira, Freek Daams, Donald L. van der Peet

**Affiliations:** 1 Amsterdam UMC Location Vrije Universiteit Amsterdam, Surgery, De Boelelaan 1117, Amsterdam, The Netherlands; 2 Department of Computer Science, Vrije Universiteit Amsterdam, De Boelelaan 1105, Amsterdam, The Netherlands

**Keywords:** ChatGPT, Artificial intelligence, Surgery

## Abstract

ChatGPT has brought about a new era of digital health, as this model has become prominent and been rapidly developing since its release. ChatGPT may be able to facilitate improvements in surgery as well; however, the influence of ChatGPT on surgery is largely unknown at present. Therefore, the present study reports on the current applications of ChatGPT in the field of surgery, evaluating its workflow, practical implementations, limitations, and future perspectives. A literature search was performed using the PubMed and Embase databases. The initial search was performed from its inception until July 2023. This study revealed that ChatGPT has promising capabilities in areas of surgical research, education, training, and practice. In daily practice, surgeons and surgical residents can be aided in performing logistics and administrative tasks, and patients can be more efficiently informed about the details of their condition. However, priority should be given to establishing proper policies and protocols to ensure the safe and reliable use of this model.

## Introduction

Artificial intelligence (AI) is a rapidly developing technology with many documented applications within surgery in the form of machine learning (ML) to forecast surgery duration, postsurgical complications, and surgical outcomes [1–3]. The rapid development of AI can be characterized by the introduction of ChatGPT [4], an AI-powered chatbot released by OpenAI on November 30, 2022, which utilizes natural language processing (NLP) to comprehend and respond to human language. Although ChatGPT presents itself as a potential tool for helping to improve our approach to surgery, the influence of ChatGPT within the field of surgery is largely unknown.

We, therefore, report on the current applications of ChatGPT in surgery, evaluating its workflow, practical implementations, limitations, and future prospects.

### Evolution of language models

The field of NLP has undergone remarkable advancements in recent years [5], particularly with regard to the release of ChatGPT, which can generate human-like responses to queries. In comparison, conventional NLP models, which rely on recurrent neural networks (RNNs) or convolutional neural networks (CNNs) to process and analyze language, still have difficulty generating the same level of human-like responses [6, 7]. These limitations are due to the nature and architecture of these older models, as RNNs and CNNs can only process relatively small tracts of text or in fixed-size windows, thereby limiting their ability to capture long-range interactions between words [6, 8]. For example, conventional NLP models were shown to be unable to understand the relationships between words in one complete sentence, and words at the beginning of a sentence had less influence on the output than words at the end [please check this carefully] [9].

In contrast to these older models, ChatGPT uses transformers designed to allow NLP models to manage long sequences of text through what is known as a “self-attention” mechanism [10]. This mechanism allows ChatGPT to receive large amounts of text as input data and process it in a parallel and non-sequential manner, thus making it better and more efficient at capturing the long-term relationships that exist between words, sentences, and paragraphs. Unlike conventional NLP models, which typically require labeled text data for training, ChatGPT is trained in an unsupervised manner, indicating that it is able to learn useful representations of text in large quantities, ranging from a variety of sources and dating as recently as September 2021 [4]. ChatGPT was modified on a large dataset of conversational data to improve its ability to generate human-like responses. This overcomes the laborious process of creating labeled text data for training purposes and allows it to become knowledgeable about a wide range of fields, as opposed to a model trained on texts from a specific field [11].

**Fig. 1:**
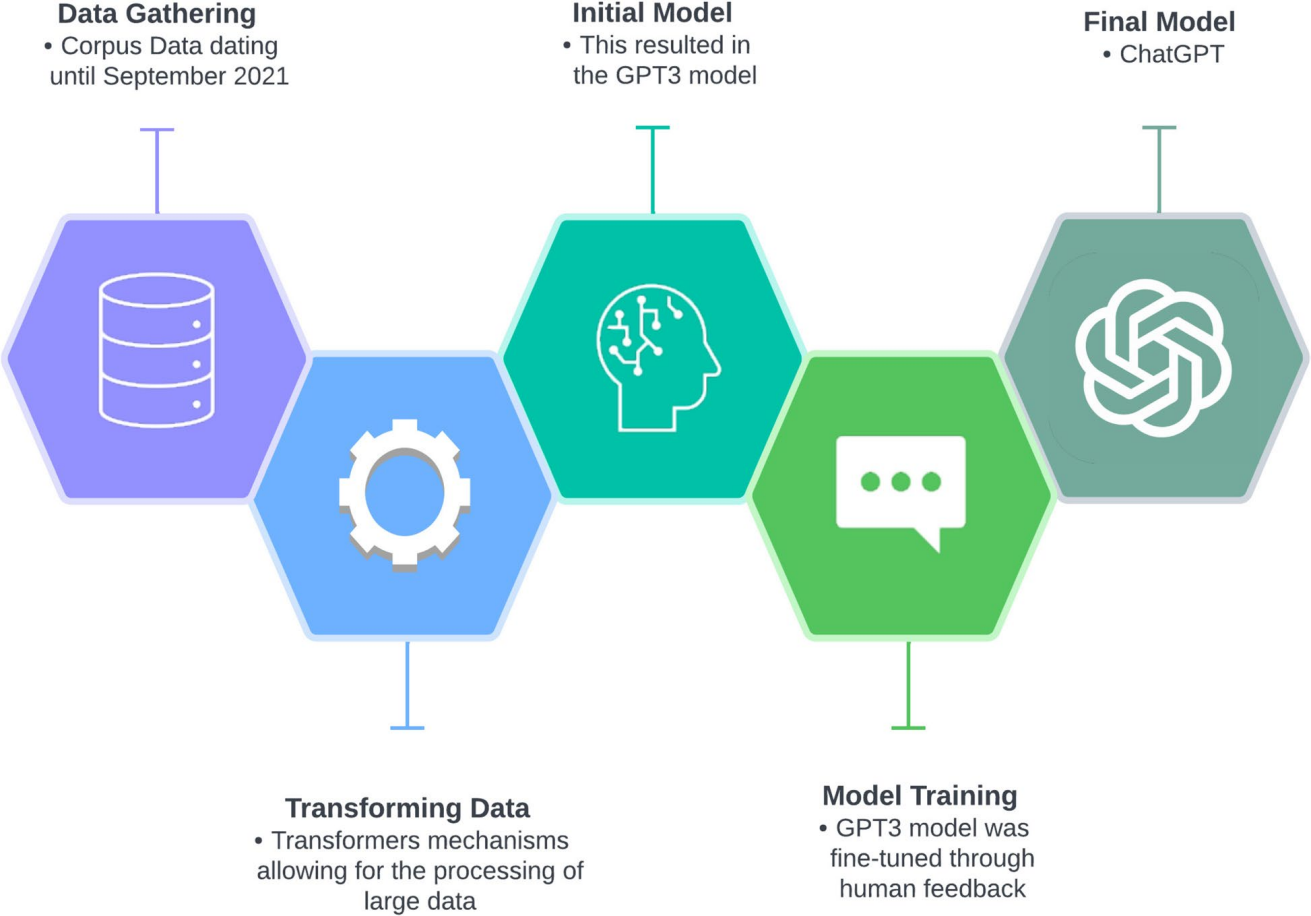
Workflow process of ChatGPT

An overview of the latest workflow process for the ChatGPT is shown in Fig. 1. Another key reason for the discrepancies that exist between ChatGPT and older NLP models in generating meaningful responses is that ChatGPT is context-aware [12]. Context awareness in NLP is the process of analyzing the current conversation and considering previous queries and responses as well as the broader context, which allows it to maintain a human-like conversation. Furthermore, the ChatGPT architecture comprises 13.5 billion parameters, making it one of the largest and most complex NLP models developed to date [4].

On March 14, 2023, GPT-4 was released by OpenAI as the newest iteration of the ChatGPT model [4]. The first key feature of GPT-4 is its ability to understand more nuanced and longer prompts than previous versions. This new model can manage prompts of up to 25,000 words, in comparison to GPT-3.5, which can only process up to 8000 words at once. GPT-4 also outperformed its predecessor in a series of examinations. In addition, GPT-4 has new multimodal features, as it can process not only text prompts but also image prompts. GPT-4 is considered safer and less likely to respond to inappropriate requests than previous iterations. Furthermore, GPT-4 has been trained on data beyond September 2021, indicating that it can answer queries more accurately, incorporating the most up-to-date knowledge [13].

### Surgical implementations

#### Research and education

Owing to its advanced analytical capabilities, ChatGPT has already demonstrated good performance in the field of research. In academic writing, ChatGPT can read research papers, summarize the content, perform analyses, and identify relevant prior work. To this end, ChatGPT has already been used to generate drafts of manuscripts, abstracts, research protocols, and research proposals [14, 15]. In addition, correcting errors and restructuring manuscripts are also some of the services that ChatGPT can perform. It has been demonstrated that ChatGPT can recommend codes for statistical analyses and simulate several model outputs to support users who are conducting such analyses [16]. With the introduction of PubMed GPT, surgical researchers could be aided in identifying academic papers of note and summarizing the most relevant paragraphs based on questions submitted to ChatGPT [17].

Surgeons and surgical residents can also use this model to keep track of the most recent advancements in surgical research. For example, a recent study illustrated how ChatGPT explains the potential benefits of AI in surgery and what is needed to fulfill its implementation in clinical practice [18]. The capabilities of ChatGPT have also been demonstrated in the field of surgical education. One study had the latest GPT-4 model take the surgical board exam, which surgical residents need to pass to complete their surgical training [19]. This exam consists of 280 complex surgical questions involving all domains of general surgery. After querying GPT-4 on all 280 surgical questions, the study discovered that the model provided correct answers to 76% of questions, demonstrating the capability of this model to understand even complex surgical information.

In addition, ChatGPT may be able to serve as a learning platform to improve residents’ surgical skills. Its ability to recognize intraoperative surgical steps and analyze trends in assessment scores could provide personalized feedback to residents to optimize their learning style. During virtual reality-based simulation training, ChatGPT might be able to evaluate residents’ performance by classifying their skill levels during the task. The integration of ChatGPT in virtual reality has the potential to enable an engaging experience in which questions can be asked interactively, and practical feedback can be received on-site to improve one’s surgical technique during simulation training [20].

#### Patient-centered perspectives

Theoretically, by summarizing the most relevant patient history, physical findings, and laboratory results, ChatGPT may be able to help surgeons and surgical residents prepare medical notes and revise their differential diagnoses. In addition, it may be possible to recommend relevant physical tests and treatment plans based on data extracted from electronic health records. A recent study emphasized the potential utility of ChatGPT for informing patients of their condition by generating clinical letters to hypothetical patients with skin cancer. On a scale of 0 to 10, ChatGPT-produced letters received a median score of 7 for correctness and readability [21]. The capabilities of this model can be harnessed to decrease the logistics and administrative workload in clinical practice, such as by, for example, explaining the surgical procedure to patients or focusing on answering patients’ questions. In turn, surgeons and surgical residents can use this new free time to improve the quality of their consultations.

In addition to supporting administrative tasks, ChatGPT can be used to increase healthcare literacy for patients [22]. ChatGPT can summarize complex diagnoses, explain medical terms, and interpret laboratory results in a way that can be understood by patients without any medical knowledge. A recent study illustrated how ChatGPT could be used as a decision-making tool in surgery. One study assessed ChatGPT’s ability to outline the best treatment plan for patients with a primary diagnosis of early breast cancer by benchmarking its answers with their own treatment recommendations [23]. ChatGPT was prompted by the patient age, breast cancer TNM status, estrogen receptor expression, progesterone receptor expression, Her2Status, Ki-67, and genetic mutations. In response, ChatGPT recommended treatment modalities, such as surgery, radiotherapy, endocrine therapy, chemotherapy, and antibody treatment. ChatGPT also provided additional information on hereditary risk factors and evaluated the costs and benefits of the recommended therapy.

#### Clinical practice

To illustrate the use of ChatGPT in surgical preparations, prompts were formulated and submitted to the latest GPT-4. Table 1 demonstrates how ChatGPT can recommend a stepwise approach to prevent postoperative complications after appendectomy. The interactive nature of ChatGPT enables the user to ask additional questions, such as, “What is the best surgical technique for appendectomy?” In addition, ChatGPT addresses the options for open and laparoscopic surgery, even explaining the indications and benefits of these approaches. Furthermore, when asked to outline the necessary steps of laparoscopic surgery, it provides an accurate overview of these steps while simultaneously elaborating on why and how each step is performed. ChatGPT even evaluates the prognosis after an appendectomy procedure and reports the factors that could affect this prognosis.

**Table 1:** Examples of using GPT-4 in surgical practice

**Prompt by clinician**	**What is the best approach to optimizing postoperative complications after appendectomy?**
GPT-4	The best approach to optimizing postoperative complications after appendectomy involves a combination of preoperative assessment, surgical technique, and postoperative care
	Preoperative assessment: before the surgery, a thorough preoperative assessment should be performed to identify patients who are at increased risk of complications. This assessment should include a detailed medical history, physical examination, and laboratory investigations to identify any preexisting medical conditions that may affect the surgery
	Surgical technique: during the surgery, the surgeon should use the appropriate surgical technique to minimize the risk of complications. This includes using minimally invasive techniques, such as laparoscopy, when appropriate. The surgeon should also use appropriate surgical instruments and take care to avoid injury to surrounding organs and tissues
	Postoperative care: after the surgery, appropriate postoperative care is critical to optimize outcomes and prevent complications. This includes appropriate pain management, early mobilization, and monitoring for signs of complications, such as fever or wound infection. Patients should also be instructed to avoid strenuous physical activity and heavy lifting for several weeks after the surgery
	In addition, patients should be educated about the signs and symptoms of potential complications, such as wound infections, and advised to seek medical attention promptly if these occur
	Overall, a multidisciplinary approach involving close collaboration between the surgeon, anesthesiologist, and nursing staff is critical to optimizing outcomes and minimizing the risk of complications after appendectomy
**Clinician**	**What is the best surgical technique for appendectomy?**
GPT-4	The best surgical technique for appendectomy depends on the individual patient’s condition and the surgeon’s experience and expertise. In general, there are two main approaches to performing an appendectomy: open surgery and laparoscopic surgery
	Open surgery: open surgery involves making a single incision in the lower right abdomen to remove the appendix. This technique is generally reserved for patients who are not suitable candidates for laparoscopic surgery due to factors such as obesity, pregnancy, or prior abdominal surgery. Open surgery may also be necessary if the appendix has ruptured or if there is suspicion of cancer
	Laparoscopic surgery: laparoscopic surgery, also known as minimally invasive surgery, involves making several small incisions in the abdomen and inserting a laparoscope (a thin, flexible tube with a camera and light) and other surgical instruments to remove the appendix. This technique is associated with less pain, a shorter hospital stay, and a faster recovery time compared to open surgery
	In general, laparoscopic surgery is the preferred surgical technique for most patients undergoing appendectomy. However, the decision about which technique to use should be based on the individual patient’s condition and the surgeon’s expertise. It is important to discuss the benefits and risks of each technique with the surgeon before the surgery to make an informed decision
**Clinician**	**For laparoscopic surgery, could you outline the detailed steps of this procedure?**
GPT-4	Sure, here is a general outline of the steps involved in a laparoscopic appendectomy:
	1. Anesthesia: the patient is placed under general anesthesia
	2. Incisions: the surgeon makes 3–4 small incisions in the abdomen
	3. Insertion of the laparoscope: the laparoscope, which is a thin, flexible tube with a camera and light source, is inserted through one of the incisions
	4. Carbon dioxide gas: carbon dioxide gas is used to inflate the abdomen, which creates space for the surgeon to work
	5. Insertion of surgical instruments: other surgical instruments are inserted through the other incisions, including a grasper, a dissector, and a stapler or scissors
	6. Identification of the appendix: the surgeon uses the laparoscope to identify the appendix and determine its location and any signs of inflammation or other abnormalities
	7. Dissection and removal of the appendix: the surgeon uses the surgical instruments to carefully dissect the appendix from the surrounding tissues and vessels, and then removes it
	8. Inspection of the surgical site: the surgeon inspects the surgical site to ensure that there is no bleeding or other complications
	9. Closure of incisions: the incisions are closed using sutures or surgical glue
	10. Recovery: the patient is taken to the recovery room to wake up from the anesthesia, and then is monitored for a period of time before being discharged from the hospital
	It is worth noting that these steps may vary slightly depending on the patient’s individual situation and the surgeon’s preference. In addition, some surgeons may choose to use a different set of surgical instruments or techniques to perform the surgery
**Clinician**	**What is the prognosis after an appendectomy procedure?**
GPT-4	After an appendectomy procedure, the prognosis is generally excellent. Appendectomy is a commonly performed surgery, and the vast majority of patients experience a full recovery without any long-term complications. Here are some key points regarding the prognosis:
	1. Recovery time: most patients recover fully within a few weeks after the surgery. The exact recovery time can vary depending on individual factors, such as the surgical approach (laparoscopic or open) and the presence of any complications
	2. Complications: while complications after appendectomy are relatively rare, they can occur. The overall rate of complications is low, but it is important to be aware of potential risks. Some possible complications include surgical site infection, abscess formation, wound dehiscence (opening of the incision), postoperative ileus (temporary disruption of normal bowel function), and in rare cases, injury to surrounding organs during the procedure
	3. Infection: infection of the surgical site or the abdomen can occur after an appendectomy. Prompt recognition and treatment of infections are crucial to prevent further complications. Antibiotics may be prescribed to treat or prevent infections
	4. Recurrence: recurrence of appendicitis is extremely rare after an appendectomy. The removal of the appendix eliminates the risk of future appendicitis. However, in some cases, other conditions can cause similar symptoms, so it is important to seek medical evaluation if new abdominal pain or other concerning symptoms occur
	5. Long-term effects: in general, an appendectomy does not lead to any long-term health issues. The appendix is a vestigial organ, and its removal does not affect digestion or overall health. Patients can typically resume their normal activities and diet without any restrictions
	It is important to note that the prognosis can vary depending on individual factors, such as the patient’s overall health, the presence of any underlying conditions, and the occurrence of complications. Your healthcare provider will be able to provide you with specific information and guidance based on your individual situation

Recently, AI has been shown to be capable of navigating during surgery. By analyzing the textures of relevant structures on images, such as X-ray or CT scans, three-dimensional models of the surgical area can be built along with major landmarks [24]. During surgery, this property is used to autonomously segment and label anatomical structures to facilitate accurate navigation by the operator. The latest GPT-4 model accepts images, analyzes important landmarks, and provides an accurate interpretation of the key components and related functions [4]. This capability could be useful for navigation during surgery. However, the extent of this capability is still being explored, as no studies have used GPT-4 in the operating room.

Although ChatGPT can produce comprehensive and relevant answers to surgical prompts, whether or not these answers are sufficiently accurate in comparison to the surgeon’s appraisal remains unclear. One study evaluated this comparison by gathering 151 surgical questions from the American Society for Metabolic and Bariatric Surgery (ASMBS) to serve as prompts for ChatGPT [25]. These included the questions most frequently asked by patients who underwent bariatric procedures. The 151 questions covered the following domains of efficacy and safety of bariatric procedures, preoperative preparations, postoperative complications, and lifestyle adaptations. All ChatGPT responses were independently graded by two bariatric surgeons as “comprehensive,” “correct but inadequate,” “some correct and some incorrect,” or “completely incorrect.” The study found that 87% of responses ChatGPT generated were “comprehensive,” indicating that bariatric surgeons had no important information to add.

### Limitations

Despite the promising potential of this model, several concerns should be addressed before its implementation.

Notably, ChatGPT has the possibility of producing factually incorrect outputs, which can be generated when the trained dataset is insufficient to answer a prompt or question. This phenomenon is described as an “artificial hallucination” and is seldom reported in chatbots [26]. However, such misleading outputs, e.g., in medical notes, could have severe consequences for patient treatment. “Artificial hallucination” was demonstrated in a study in which references were fabricated by ChatGPT by asking them to write several medical papers [27]. Almost half of the references were fabricated, whereas the remaining references were authentic but still inaccurate, and only a small percentage of references were authentic and accurate. Furthermore, ChatGPT cannot function independently at present, without the guidance of humans [28]. It is clear that the level of surgical conception and expertise of ChatGPT is inferior to that of actual surgeons and surgical residents. Surgeons can make decisions in real time in unexpected circumstances, and ChatGPT cannot replace this ability. Therefore, ChatGPT is not at the level of being able to replace surgeons in surgical decision-making, instead being more appropriate for use as a guidance tool. Ultimately, the surgeon must take responsibility for any mistakes that were influenced by interaction with ChatGPT, as using this model could have severe consequences, such as causing undue complications and mortality.

Another point of concern is that, because ChatGPT will use patient data, such as their histories, laboratory results, and diagnoses, this information will be stored automatically. These sensitive data will then be rendered susceptible to unauthorized access, re-identification, or data leakage [29]. In the context of patient safety, these valuable data should be collected and processed in a secure and anonymized manner. It should be emphasized that these data should only be used for their intended purpose. Furthermore, it is vital to provide transparency regarding the use of patient data. Large amounts of data were used at a high rate during the training phase of ChatGPT. However, this can lead to negligence concerning patient autonomy, as there are no strict rules concerning informed consent regarding the utilization of patient data. Any form of data leakage or misuse could have severe ethical consequences; however, as no consensus has yet been achieved regarding who is responsible for such consequences, the question remains whether clinicians should be fully accountable for any errors that occur concerning the use of ChatGPT. This ambiguity could lead to potential medical-legal issues [30]. Therefore, users of ChatGPT should be aware of these limitations and understand that this model functions best as a supportive tool provided with proper guidance and surgical expertise.

### The future of ChatGPT

In the future, the functionalities of ChatGPT should be extended, and we expect the capabilities of this model to be increasingly intimately integrated into the daily practice of surgery. A new function may include the examination and processing of visual data, such as a photograph of an infected wound. As the latest GPT-4 model can also analyze images and videos, it will be possible to train GPT-4 on datasets consisting of surgical photos and videos [4]. The accuracy of GPT-4 in recognizing image objects and actions depends largely on the availability and accessibility of high-quality training data. Regarding the utility of GPT-4 versus other AI imaging models that have been trained on CT images and endoscopic images for diagnostic purposes, it may be preferable to still use those other models, as the type of data they have been trained on is not a black box [31, 32]. In addition, medical data and images are highly inaccessible owing to the protection of patient privacy; therefore, it is unlikely that GPT-4 has access to this type of data [33]. However, one study comparing GPT-3 and GPT-4 showed that language models were capable of labeling metastatic disease through text prompts of CT reports of lung cancer patients. By extracting lesion diameters and assessing oncologic progression without the need for CT images, GPT-4 showed higher accuracy in extracting lesion parameters, identifying metastatic disease, and generating correct labels for oncologic progression than the GPT-3 model [34]. GPT-4 could allow for new possibilities for surgery and may eventually support surgeons and surgical residents in their clinical decision-making to enhance patient care. However, it is important to emphasize the need to maintain critical thinking and improve knowledge, as this innovation should serve as a supportive tool for administrative tasks and not a replacement.

To facilitate the implementation of this innovation, efforts should be made to address the current challenges associated with ChatGPT. If ChatGPT is to be used to establish electronic health records, patients should be educated and informed about this procedure, with informed consent obtained before their data are included. In addition, to address medical-legal issues, clear policies and protocols should be established in every hospital to ensure the proper security and privacy of patient data. Formal guidelines should be formulated concerning the use of ChatGPT in drafting manuscripts. It should be clear that ChatGPT can be used to gather scientific information and restructure manuscripts, but generating a complete manuscript with ChatGPT should be considered a misuse of this innovation.

In addition, there is no specific tool to evaluate the reliability of ChatGPT answers, although the Ensuring Quality Information for Patients (EQIP) tool could be used until a proper guideline for ChatGPT is established. The EQIP tool evaluates the quality of any digital information by assessing the domains of content, identification, and structure of the information, emphasizing the completeness and accuracy of the information [35]. Another solution could be to assess the overlap between local hospital guidelines and the answers provided by ChatGPT. Interrater agreement could serve as a measure of the validity of ChatGPT answers [36]. An alternative possibility could be the use of statistics, such as correlation coefficients, to evaluate the compliance between the output and true observations [37]. However, specific and comprehensive tools for AI chatbots should be developed to systematically evaluate the reliability and validity of their answers. These tools can be developed by following a stepwise approach, similar to the establishment of the PROBAST Tool for AI models [38]. In that approach, literature reviews were performed, and surveys were completed by experts to determine validity items, followed by consensus meetings to establish definitive guidelines. Once these challenges have been overcome, ChatGPT can function as a valuable supportive tool in surgical research, education, training, and practice.

## Conclusion

In conclusion, ChatGPT demonstrated its capabilities in the areas of surgical research, education, training, and practice. Surgeons and surgical residents were supported in writing manuscripts, making medical notes, and preparing for surgery. In addition, patients were able to be informed more comprehensively and efficiently using ChatGPT. However, it is essential to consider the concerns associated with the use of ChatGPT. By taking appropriate measures, ChatGPT may be able to serve as a useful tool in surgery by enhancing human capabilities.
